# Theoretical Local Buckling Behavior of Thin-Walled UHPC Flanges Subjected to Pure Compressions

**DOI:** 10.3390/ma14092130

**Published:** 2021-04-22

**Authors:** Jeonghwa Lee, Seungjun Kim, Keesei Lee, Young Jong Kang

**Affiliations:** 1 Future and Fusion Laboratory of Architectural, Civil and Environmental Engineering, Korea University, Seoul 02841, Korea; qevno@korea.ac.kr; 2 School of Civil, Environmental and Architectural Engineering, Korea University, Seoul 02841, Korea; rocksmell@korea.ac.kr; 3 Department of Urban Infrastructure Research, Seoul Institute of Technology, Seoul 03909, Korea; kslee@sit.re.kr

**Keywords:** ultra-high-performance concrete, UHPC, local buckling, stability, thin-walled flange, nonlinear analysis

## Abstract

To enhance structural performance of concrete and reduce its self-weight, ultra-high-performance concrete (UHPC) with superior structural performance has been developed. As UHPC members with 180 MPa or above of the compressive strength can be designed, a rational assessment of thin-walled UHPC structural member may be required to prevent unexpected buckling failure that has not been considered while designing conventional concrete members. In this study, theoretical local buckling behavior of the thin-walled UHPC flanges was investigated using geometrical and material nonlinear analysis with imperfections (GMNIA). For the failure criteria of UHPC, a concrete damaged plasticity (CDP) model was applied to the analysis. Additionally, an elastic-perfectly plastic material model for steel materials was considered as a reference to establish differences in local buckling behavior between the UHPC and steel flanges. Finite element approaches were compared and verified based on test data in the literature. Finally, this study offers several important findings on theoretical local buckling and local bending behavior of UHPC flanges. The inelastic local buckling behavior of UHPC flanges was mainly affected by crack propagation due to its low tensile strength. Based on this study, possibility of the local buckling of UHPC flanges was discussed.

## 1. Introduction

Concrete has been widely used as a construction material for constructing infrastructures such as bridges, tunnels, buildings, pavements, etc. Although conventional concrete is weaker than steel, it has been among the most popular construction materials because of its high durability, low maintenance cost, excellent workability, and good fire resistance. Conventionally, concrete members are reinforced using steel rebars for enhanced resistance capacity to compensate for their low tensile strength. The failure mode of reinforced concrete (RC) structural members was mainly controlled by the yield of the reinforced rebars. The compressive failure or instability of concrete members was not a major concern in concrete structures before ultra-high-performance concrete (UHPC) with 180 MPa or above of compressive strength was developed.

Since ultra-high-performance concrete (UHPC) has been developed to enhance the structural performance of conventional concrete structures and reduce their self-weight, it has been widely used in various applications such as beams, columns, connections, decks, and long-span bridge members in construction. The term UHPC was first introduced by Larrard [1] in 1994; UHPC has been continuously developed in Europe, North America, and Asia. In the 2000s, reactive powder concrete was developed by Bouygues of France [2], Ductal^®^ in North America [3], and CEMTEC [4] at LCPC in France. Furthermore, K-UHPC [5] was developed by the Korea Institute of Construction Technology in South Korea. Conventionally, UHPC is characterized by high compressive and tensile strengths. It features a compressive strength greater than or approximately equal to 180 MPa, which is achieved by reducing the water–cement ratio and curing at high temperatures. It is also characterized by a high tensile strength, which is approximately 10 MPa or above with ductility, owing to the mixing of steel fibers in the concrete mixture. Design standards applicable to UHPC have been introduced and applied in France, Japan, Australia, and South Korea [6,7,8,9]. 

**Figure 1:**
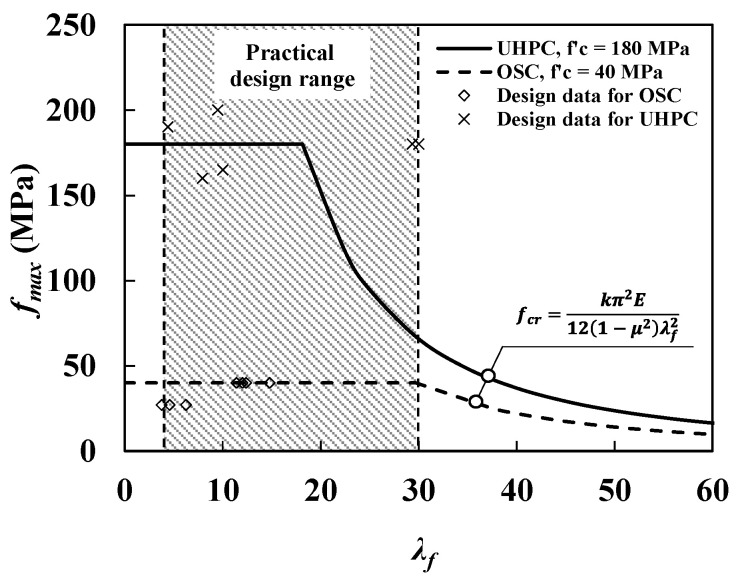
A comparison between theoretical elastic local buckling strength ($f_{cr}$) with respect to the slenderness ratio ($\lambda_{f}$) and practical design range of UHPC and ordinary strength concrete (OSC) for compression bridge decks (data from reference [23]).

Since the compressive strength of UHPC exceeds 180 MPa, which is similar to that of conventional structural steels, designing a thin-walled concrete member can be possible. It has been reported that the flange width-to-thickness ratios ($\lambda_{f}$) in the current design ranges of the bridge deck geometry range from 4 to 30 for outer UHPC bridge decks as indicated in Figure 1. In general, the bridge deck with ordinary strength concrete (OSC) under 40 MPa of compressive strength has no possibility of buckling failure modes based on bridge data of OSC as shown in Figure 1. However, for the UHPFRC bridge decks with 180 MPa of compressive strength, the possibility of buckling failure modes may drastically increase due to its high compressive strength, which is approximately the same level in compressive strength as steel flange’s (typical yield strength of steel flanges are ranged from 230 to 315 MPa). Furthermore, the slenderness ratio of practically designed outer decks can be increased up to 30, which may be considered as a highly slender flange section. Therefore, a rational assessment of the buckling stability of thin-walled UHPC structural members, which has not been considered in conventional concrete member designs, is now necessary for avoiding unexpected buckling failure and for careful design practice. Although realistic UHPFRC flanges are frequently stiffened for prestressing systems and designed based on effective-width approaches that produce conservative deck designs, it is important to verify the likelihood of local buckling phenomena in thin-walled UHPFRC members to secure more reasonable, safe, and efficient design practices for the application of the thin-walled UHPFRC members.

The instability of concrete members has been rarely considered as a controlling failure mode in the previous studies regarding the lateral instability of concrete girders only. Pioneering studies concerning the lateral instability of concrete girders were conducted from 1950 to 1960. Marshall [10], Hansell and Winter [11], Sant and Bletzacker [12], and Massey [13] have demonstrated the phenomena of the lateral torsional buckling in concrete girders with rectangular sections and established the corresponding theoretical backgrounds through experimental studies. Since then, several studies have focused on the lateral torsional buckling of RC girders [14,15,16,17,18]. Additionally, ACI 318-14 [19], a design specification for concrete girders, stipulates that the slenderness limit of the girders should be considered to prevent their lateral torsional buckling.

**Figure 2:**
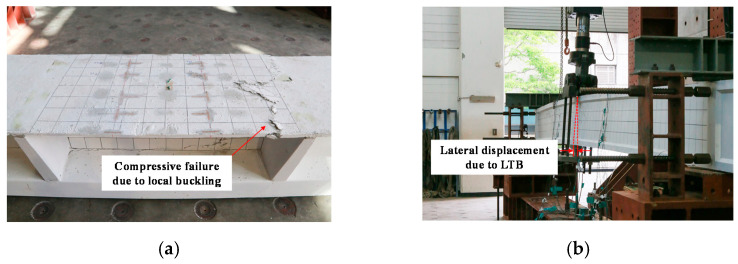
Examples of buckling failure in concrete slender members: (**a**) local buckling of thin-walled ultra-high performance concrete (UHPC) flanges (a test specimen conducted from reference [22]) and (**b**) lateral-torsional buckling (LTB) of slender UHPC I-girder (test specimen conducted from reference [21]).

Recently, studies on the structural instability of UHPC compression and flexural members have been conducted. Illich et al. [20] conducted an experimental study on UHPC column specimens that were subjected to compressive forces to observe the global buckling of slender columns. Lee et al. [21] conducted an experimental study on slender UHPC girder specimens and established a design equation for elastic and inelastic LTB strength while considering nonlinear material properties and the effective moment of inertia. Lee [22] investigated the experimental tests and numerical evaluations of the local buckling in UHPC thin-walled I-girder flanges. Additionally, they presented moment capacity equations considering the local buckling phenomenon [23]. The buckling failures of the slender concrete members are illustrated in Figure 2.

Modern concrete designs such as long-span bridges employ thin-walled, and slender UHPC concrete structural members to reduce the self-weight of the entire structure. In general, the outer flange may be more likely to undergo instability, because a flange supported by one edge has a lower buckling coefficient (k) than those supported by two edges. Considering these perspectives, the buckling instability of thin-walled and slender concrete members may possibly emerge as an important engineering issue and its significance may increase as the strength of concrete increases.

In this study, the local buckling strength of UHPC flanges was evaluated based on geometrical and material nonlinear finite element (FE) analysis with imperfections (GMNIA) by considering two different boundary conditions. For material failure criteria, a concrete damaged plasticity (CDP) material model with a tensile strength of approximately 5% compared to its compressive strength was applied to the UHPC flanges. An elastic-perfectly-plastic material model (plastic material model) that considers equal yield in compressive and tension was adopted as a reference material model to establish the basic differences in local buckling behavior between the UHPC and steel flanges. The presented finite element approaches for analyzing the local buckling of UHPC flanges were compared and verified based on test data in the literature [22]. This study offers several important findings on the inelastic local buckling and local bending behavior of UHPC flanges. It was concluded that the inelastic local buckling behavior of UHPC flanges was mainly affected by crack propagation because of its low tensile strength. Further, when the compressive strength and slenderness ratio are equal, the post-buckling strength of UHPC flanges has a marginal effect, as compared to that of steel flanges. Based on this study, possibility of the local buckling of UHPC flanges was discussed.

## 2. Material Properties of UHPC

The UHPC exhibited a compressive strength of approximately 180 MPa and an elastic modulus of 45,000 MPa, achieved by reducing the water–cement ratio without a coarse aggregate. In addition, high-temperature steam curing can be employed to improve the robustness of the UHPC microstructure. The tensile strength and ductility of UHPC can be improved by adding approximately 2% of steel fibers to the concrete admixture. Examples of a conventional UHPC mix design are listed in Table 1.

Graybeal [3] presented the compressive strength and strain relationships of the ascending branch for estimating UHPC compression material behavior. In this model, the ultimate strain of UHPC can be estimated as 0.0042 under a compressive strength of 180 MPa. The compressive strength and strain relationships are presented as follows:

(1) $$f_{c}=\varepsilon_{c}E\left(1-\alpha \right)$$

(2) $$\alpha =ae^{\frac{\varepsilon_{c}E}{bf_{c}^{\prime }}}-a$$

where α is the percentage decrease in stress compared to the linear elastic predicted stress, $\varepsilon_{c}$ is the compressive strain in concrete corresponding to $f_{c}$, and a, b are constants related to curing.

**Figure 3:**
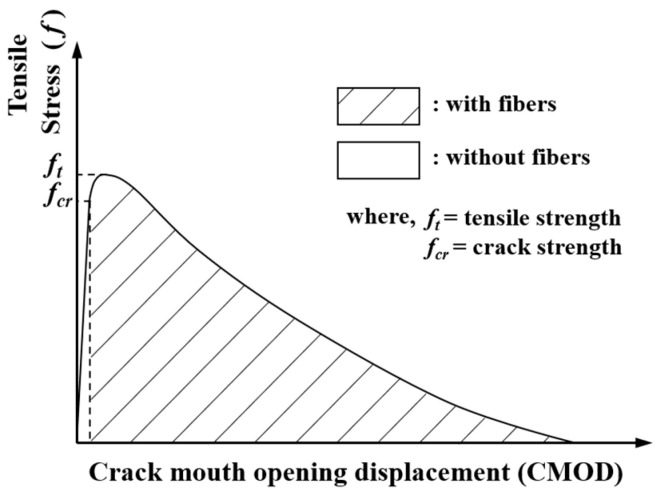
Typical tensile behavior of UHPC.

The tensile strength of UHPC is considerably higher than that of ordinary concrete. Additionally, greater ductility and tensile strength, attributed to steel fibers, can be observed after the first cracking. Therefore, the ultimate tensile strength ($f_{t}$) of UHPC can be defined as the post-peak strength ($f_{peak}$) that develops after the crack strength ($f_{cr}$) induced by initial cracking. The conventional tensile behavior of UHPC is illustrated in Figure 3. Graybeal [3] proposed tensile strength equations for UHPC while considering the compressive strength, as shown in Equation (3). The coefficients 7.8 and 8.3 in Equation (3) represent the lower and upper bounds of the tensile strength, respectively, considering steam curing. In the case of untreated specimens, the coefficient was assumed to be 6.7.

(3) $$f_{t}=7.8\sqrt{f_{c}^{\prime }}\;\mathrm{or}\;8.3\sqrt{f_{c}^{\prime }}\;\mathrm{in}\;\mathrm{psi}\;\mathrm{unit}$$

where, $f_{t}$ is the tensile strength of UHPC and $f_{c}^{\prime }$ is the compressive strength of UHPC.

The relationship between the compressive strength and elastic modulus of concrete can be estimated using the following equation [3]:

(4) $$E_{c}=49,000\sqrt{f_{c}^{\prime }}\;\mathrm{in}\;\mathrm{psi}\;\mathrm{unit}$$

where $E_{c}$ is the elastic modulus of the UHPC and $f_{c}^{\prime }$ is the compression strength of the UHPC.

Ma et al. [24] presented an equation for the modulus of elasticity based on compressive strength, as follows:

(5) $$E_{c}=525,000{\left(\frac{f_{c}^{\prime }}{10}\right)}^{1/3}\;\mathrm{in}\;\mathrm{psi}\;\mathrm{unit}$$

## 3. Theoretical Elastic Local Buckling Strength of Thin-Walled UHPC Flanges

For thin-walled flange members with high yield strengths, it is important to determine the local buckling strength to avoid unexpected brittle failure before yielding. Although the stress acting on the cross-section of the flanges does not reach its yielding stress, a thin-walled flange member with a large slenderness ratio may be unstable with a large out-of-plane deformation, which can dominate the overall strength instead of the yielding strength. It implies that the geometric characteristic strength affected by the width to thickness ratio (slenderness ratio, $\lambda_{f}$), in which $\lambda_{f}$ can be calculated by half width divided by thickness ($b_{f}/2t_{f}$) for the flange supported by one edge or width divided by thickness ($b_{f}/t_{f}$) for the plate supported by two edges, becomes the governing strength for the thin-walled flange sections. This type of buckling failure frequently occurs in thin-walled flange members, including steel, and FRP flanges, which are characterized by high yielding or rupture strengths.

**Figure 4:**
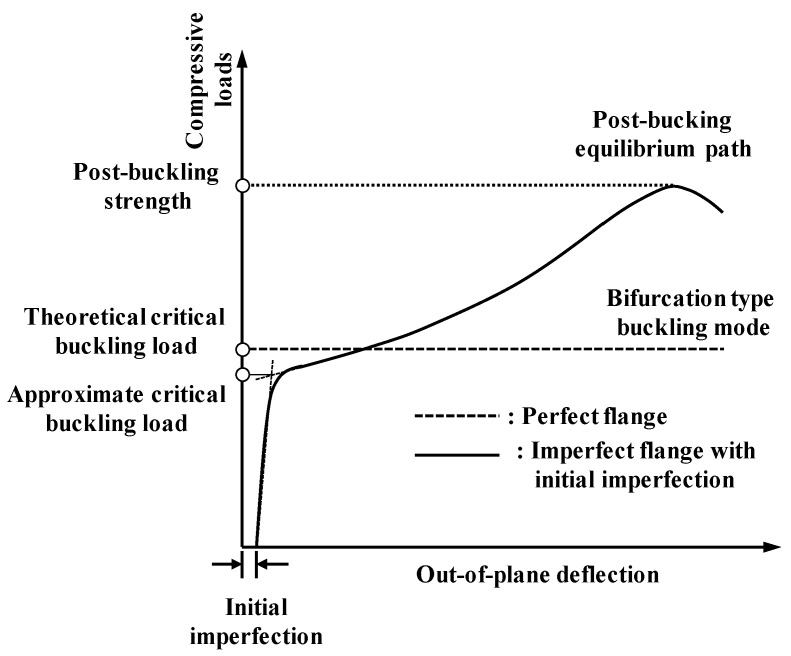
Typical local buckling behavior of thin-walled members in perfect and imperfect flanges.

Figure 4 shows the typical equilibrium path and critical buckling strength for the perfect and imperfect flanges. In the critical local buckling state of the flange within the elastic ranges, the stiffness of the flange subjected to axial compression loads theoretically becomes zero. This is called bifurcation-type buckling modes (refer to Figure 4). The elastic local buckling strength of the flange can be calculated using the theoretical buckling strength equation defined in Equation (6). The theoretical elastic local buckling strength is affected by the modulus of elasticity (E), width-thickness ratio ($b_{f}/2t_{f}$ or $b_{f}/t_{f}$), Poisson’s ratio (μ), and elastic buckling coefficient (k):

(6) $$f_{cr}=\frac{k\pi^{2}E}{12\left(1-\mu^{2}\right){\left(\lambda_{f}\right)}^{2}}$$

where $f_{cr}$ is the elastic local buckling strength, k is the elastic buckling coefficient that represents the support conditions, E is the modulus of elasticity, μ is the Poisson’s ratio, and $\lambda_{f}$ is the slenderness ratio of the flanges.

The buckling of flanges is strongly influenced by the type of load and boundary conditions. In general, the design elastic buckling coefficient ($k_{c}$) of a steel flange supported by one edge can be determined using web constraints, as follows [25]:

(7) $$k_{c}=\frac{4}{\sqrt{\frac{H}{t_{w}}}}\;\left(0.35\leq k_{c}\leq 0.76\right)$$

where $k_{c}$ is the design elastic buckling coefficient, H is the web height, and $t_{w}$ is the thickness of web panels.

In thin-walled UHPC flanges, the UHPC is a highly complex quasi-brittle heterogeneous material with different mechanical properties under tension and compression, whereas steel is a homogeneous material with equal compressive and tensile strengths and high modulus of elasticity. As UHPC contains different types of materials, including aggregates, sands, and cement, the modulus of elasticity and Poisson’s ratio are considerably lower than those of steels. These differences in material property could lead to a different elastic buckling strength than that of steel flanges.

**Figure 5:**
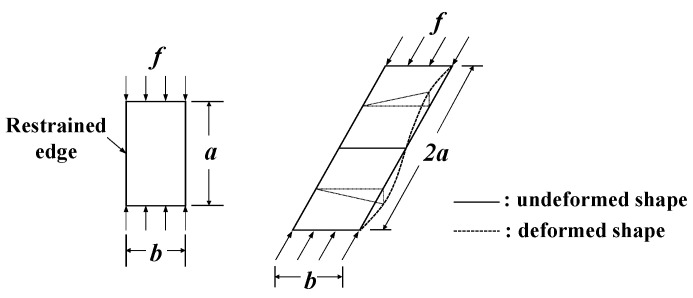
Outstanding flange under edge compression.

According to Lundquist and Stowell [26], the elastic buckling strength of a uniformly compressed flange with simply supported edges (see Figure 5) is determined using the boundary conditions, slenderness ratio, modulus of elasticity, and Poisson’s ratio. The elastic buckling coefficients (k) can be calculated using Equation (8).

(8) $$k=\frac{2}{\pi^{2}}\left\{\frac{\begin{array}{c} 1-\mu +\frac{1}{6}{\left(\frac{\pi b}{\lambda }\right)}^{2}+\frac{\varepsilon }{2}\left[\frac{c_{1}}{2}{\left(\frac{\pi b}{\lambda }\right)}^{2}+c_{2}+\mu c_{3}\right]\\ +\frac{\varepsilon^{2}}{4}\left[\frac{c_{4}}{2}{\left(\frac{\pi b}{\lambda }\right)}^{2}+\frac{c_{5}}{2{\left(\frac{\pi b}{\lambda }\right)}^{2}}+c_{6}-\mu c_{7}\right]+\frac{\varepsilon }{2{\left(\frac{\pi b}{\lambda }\right)}^{2}} \end{array}}{\frac{1}{3}+\frac{c_{8}\varepsilon }{2a_{3}}+\frac{c_{9}\varepsilon^{2}}{4a_{3}^{2}}}\right\}$$

where k is the non-dimensional elastic buckling coefficient that depends on the conditions of the edge restraint and shape of the flange, μ is Poisson’s ratio, b is the width of the flange, a is the effective buckling length, m is the number of buckling modes, λ is a parameter obtained by dividing a with m (λ=a/m), ε is the restrain coefficient $\left(=4S_{0}b/D\right)$ (ε=0; Hinged condition, ε=∞; Fixed condition), $S_{0}$ is the stiffness per unit length of the elastic restraining medium or the moment required to rotate a unit length of the elastic medium through one-fourth radian, D is the flexural rigidity of the flange per unit length $\left(Et^{3}/\left(12\left(1-\mu^{2}\right)\right)\right)$, and $c_{1}$–$c_{9}$ are the coefficients.

**Figure 6:**
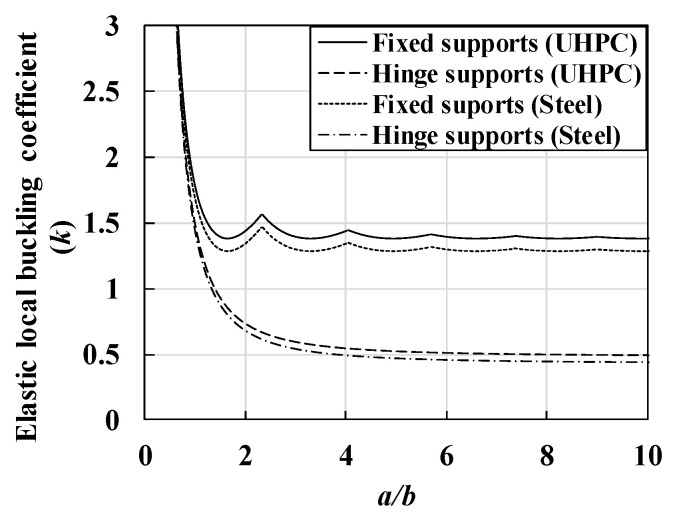
Comparison of elastic local buckling coefficient (*k*) for UHPC and steel.

**Figure 7:**
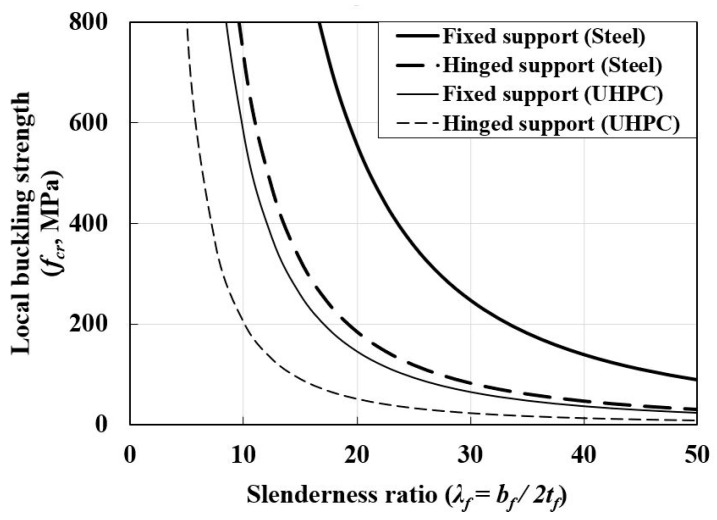
Comparison of elastic local buckling strength of UHPC and steel flanges.

By substituting a Poisson’s ratio (μ) of 0.2 and 0.3 for UHPC and steel, respectively, into Equation (8), the elastic local buckling coefficient (k) of the UHPC and steel flanges according to the two boundary conditions and a/b can be obtained, as shown in Figure 6. Additionally, Table 2 shows the modulus of elasticity (E), Poisson’s ratio (μ), and minimum elastic buckling coefficient (k) of the UHPC and steel flanges calculated using Equation (8). By substituting the values listed in Table 2 in Equation (6), the theoretical elastic buckling strength of UHPC and steel flanges can be obtained, as shown in Figure 7. In Figure 7, the UHPC flange demonstrates a local buckling strength that was approximately 1/4 times lower than that of the steel flanges owing to its low modulus of elasticity (E); however, it exhibited a higher elastic local buckling coefficient (k). Based on the theoretical evaluation, the local buckling phenomenon was more likely to occur in the UHPC concrete flange than in the steel flange members under the same boundary conditions and slenderness ratios ($\lambda_{f}$).

## 4. Inelastic Local Buckling of Thin-Walled UHPC Flanges Based on FEA

### 4.1. Overview of FEA

As mentioned in Section 3, the theoretical elastic local buckling strength of the UHPC flange may be 1/4 times lower than that of the steel flanges for the same boundary conditions and slenderness ratios. This implies that the elastic local buckling of the UHPC flange is more likely to occur. Additionally, due to the different material properties of UHPC under compression and tension, considerably different inelastic local buckling behavior may be observed.

The inelastic local buckling of UHPC flanges was investigated based on geometrical and material nonlinear FE analysis with initial imperfections (GMNIA). Here, an inelastic UHPC material model, whose tensile strength was 5% of its compressive strength, based on Graybeal [3]’s equation (Equation (3)) was employed. Additionally, an elastic-perfectly plastic model, which represents equal yield strengths under compression and tension, was used to simulate conventional steel flanges as the reference models for comparisons. 

In general, the local buckling strength of flanges can be classified into the critical buckling strength and the post-buckling strength. The critical buckling strength is a theoretical, characteristic buckling strength obtained from the elastic buckling coefficient (k), which is presented in Table 2. It is also estimated based on the compressive load and out-of-deflection relationships by defining strength, where the stiffness of a structural member decreased significantly after reaching critical buckling equilibrium conditions (such as the flange with initial imperfection in Figure 4). In practice, flange members could resist additional compression loading induced by the redundancy of tensile stress until they attain post-buckling strength. The post-buckling strength was affected by the geometric and material nonlinearities when the flange section had a high slenderness ratio.

In UHPC flanges, UHPC exhibited a considerably lower tensile strength compared to its compressive strength. These significant differences in the tensile strength of UHPC flanges affected the inelastic buckling and post-buckling behaviors. Therefore, in this study, the GMNIA analysis of the UHPC flange member was conducted to investigate the differences in inelastic and post-local buckling behaviors by comparing it with the reference steel flange models. The parameters used for the GMNIA analysis are listed in Table 3.

#### 4.1.1. FEA Models

**Figure 8:**
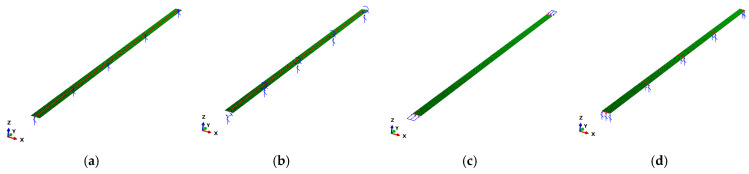
Compressive load and boundary conditions. (**a**) Hinge supports: displacement constrains along the *Z*-axis; (**b**) fixed supports: displacement constraints along the *Z*-axis and rotations along the *Y*-axis; (**c**) uniform loads at each end for the hinge and fixed condition; and (**d**) support along the *Z*-axis for the hinge and fixed conditions.

The UHPC and steel flanges were modeled with 3D shell elements (S4R) using the ABAQUS program [27]. The models featured a flange width of 1 m. Flange thicknesses were determined by varying the slenderness ratio from 2.5 to 65, as listed in Table 3. The load and boundary conditions are depicted in Figure 8. The flange models were supported by the hinge and fixed constraints along the centerline of the flange width, as shown in Figure 8a,b. Uniform compressive stresses were applied to both ends, as shown in Figure 8c. Vertical displacement of the models was prevented using equally spaced restraints along their length, as shown in Figure 8d. The length affording minimum buckling strength was determined using a convergence analysis, and it was assumed to be 40 m and 16 m for the hinged and fixed support conditions, respectively. The supported spacing along the length was in consideration of the buckling modes of the flanges, and the number of elements along the flange width and length could influence the convergence of elastic local buckling strength. Thus, the supported spacing and number of elements were determined using convergence analyses.

#### 4.1.2. Material Properties for FEA

With regard to the modulus of elasticity of UHPC, as proposed by Ma et al. [24], the lower values determined using Equation (5) were used for conservative results. Tensile strength was calculated using Equation (3), as presented by Graybeal [3].

For the simulation of UHPC, ascending branches of the compressive stress and strain relationship was determined using Graybeal’s [3] models based on Equations (1) and (2). For the descending branch, Kappos and Konstantinidis’ [28] model, which was developed for high-strength concrete, was used to simulate the brittle failure of UHPC after peak strength. Kappos and Konstantinidis’ [28] material constitutive models are shown below:

For the ascending branch,

(9) $$0<\varepsilon_{c}\leq \varepsilon_{ccl},\;\sigma_{c}=\frac{f_{cc}\frac{\varepsilon_{c}}{\varepsilon_{ccl}}\frac{E_{c}}{E_{c}-E_{cl}}}{\frac{E_{c}}{E_{c}-E_{cl}}-1+{\left(\frac{\varepsilon_{c}}{\varepsilon_{ccl}}\right)}^{\frac{E_{c}}{E_{c}-E_{cl}}}}$$

For the descending branch,

(10) $$\varepsilon_{c}>\varepsilon_{ccl},\;\sigma_{c}=f_{cc}\left[1-0.5\frac{\varepsilon_{c}-\varepsilon_{ccl}}{\varepsilon_{0.5f_{cc}}-\varepsilon_{ccl}}\right]$$

where $\varepsilon_{c}$ is the general concrete strain, $\varepsilon_{ccl}$ is the axial concrete strain at the peak stress in confined concrete, $\sigma_{c}$ is the general concrete stress, $f_{cc}$ is the maximum compressive strength of the concrete, $E_{c}$ is the tangent modulus of elasticity of the concrete, $E_{cl}$ is the secant modulus of elasticity of the concrete, $\varepsilon_{0.5f_{cc}}$ is the strain, at which the stress in plain concrete drops to $0.5f_{cc}$, and $\varepsilon_{ccl}$ is the axial concrete strain at the peak stress.

**Figure 9:**
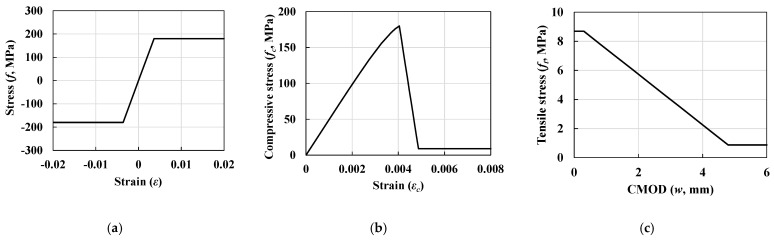
Tensile material behavior obtained via finite element analysis: (**a**) stress–strain relationship of the plastic material model for steel flanges, (**b**) compressive stress–strain relationship of the concrete damaged plasticity (CDP) material model for UHPC flanges, and (**c**) relationship between tensile strength and crack mouth opening displacement of the CDP model.

The crack displacement for tensile stress was determined using the “Design Guidelines for K-UHPC [8]”; detailed material properties are listed in Table 4. Considering the material properties for flanges, two different types of material models, namely concrete damaged plasticity (CDP) and an elastic-perfectly plastic model (plastic model), were used in the GMNIA. These two materials represent the material constitutive laws for concrete and steel, respectively (refer to Figure 9).

For the CDP model, ε, $\sigma_{b0}/\sigma_{c0}$, $K_{c}$, and ψ were assumed to be 0.1, 1.16, 2/3, and 36, respectively, where ε is the eccentricity, which is calculated as the ratio of tensile strength to the compressive strength; $\sigma_{b0}/\sigma_{c0}$ is the ratio of the strength in the biaxial state to that in the uniaxial state, $K_{c}$ is the parameter related to the failure surface of concrete, and ψ is the dilation angle.

#### 4.1.3. Initial Imperfections

In general, local buckling strength is sensitive to initial imperfections, which represent the initial defects in the flanges that occurred during the production process. In the case of typical steel flanges, the initial imperfections are mainly caused by welding heat during the fabrication process. The initial imperfections in UHPC flanges can be induced from both welding heat in the steel forms and the initial casting stage. Furthermore, methods for estimating the initial imperfections in the UHPC flanges of actual structures have not been well established. According to an experimental study by Lee [22], initial imperfections in UHPC flanges are less than the recommended value for steel flange. Therefore, in this study, the conventional initial imperfections of the steel girder flanges specified in the AWS Bridge Welding Code [29] were used. For conservative estimation, the provision of initial imperfection for a flange supported by one edge is defined as follows:

(11) $$\delta =min\left[\frac{b_{f}}{150},\frac{0.3a}{150}\right]$$

where δ is the initial imperfection, $b_{f}$ is the width of the flange, and a is the unbraced length or the length of the half-sine buckling mode of the flange.

**Figure 10:**
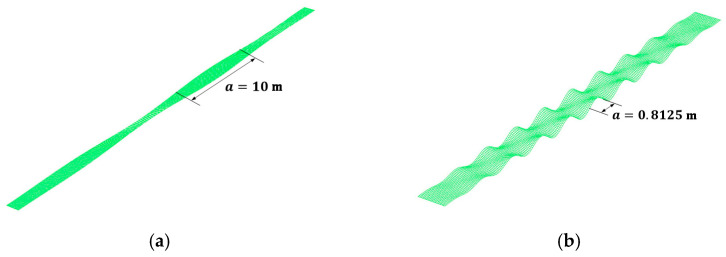
Local buckling mode shapes based on elastic buckling analysis: (**a**) hinge support condition and (**b**) fixed support condition.

**Figure 11:**
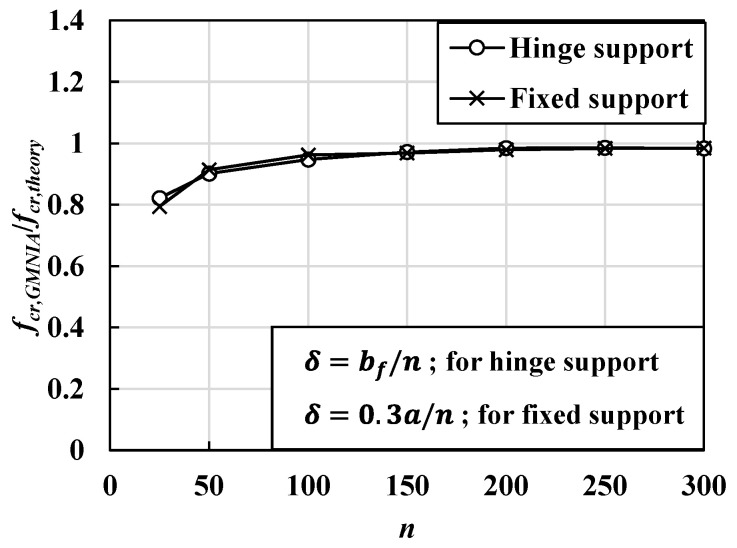
Critical buckling strength corresponding to the magnitude of imperfection (*δ*) when the slenderness ratio ($\lambda_{f}=b_{f}/2t_{f}$) is 20.

Figure 10 shows the local buckling mode shapes obtained from the elastic buckling analysis for the hinge and fixed boundary conditions. The buckling mode shape for the GMNIA was determined based on the nodal displacements of the 1st mode shapes, obtained from the elastic buckling analysis results. Figure 11 shows the elastic buckling strength corresponding to the magnitude of initial imperfections, obtained using the GMNIA. Based on the convergence analysis, the maximum initial imperfections were determined to be 6.67 mm ($b_{f}/150$) for the hinged condition and 1.625 mm (0.3a/150) for the fixed condition, based on the AWS Bridge Welding Code [29], as the convergence analysis for the two boundary conditions yields appropriate estimations.

#### 4.1.4. Definition of Critical and Post-Buckling Strength

**Figure 12:**
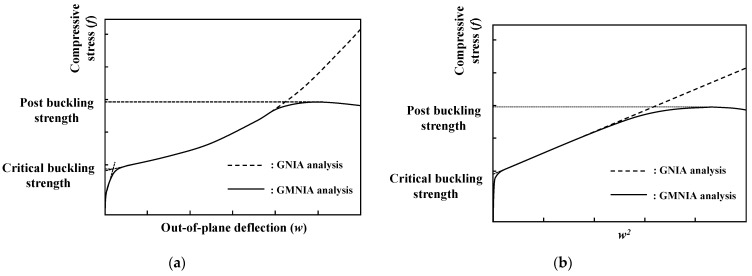
Definition of critical and post-buckling strengths based on FEA (GNIA means geometric nonlinear analysis with imperfections, and GMNIA means geometric and material nonlinear analysis with imperfections). (**a**) *f* − *w* relationship, and (**b**) *f* − *w*^2^ relationship.

The critical local buckling of flanges occurs within the elastic range during the initial stage of the compression load; out-of-plane deflection starts to be developed after the compressive stress reaches the critical buckling strength. With respect to the post-buckling behavior, the applied loading can be gradually increased until it reaches ultimate strength with large out-of-plane deformation. The ultimate strength can be defined as the post-buckling strength. Several studies have reported methods to determine critical local buckling strengths [30]. In this study, $f-w^{2}$ was used to approximate the lower boundary critical buckling strength of UHPC flanges. For a conventional f−w curve, the equilibrium path has a nonlinear relationship, as shown in Figure 12a. If the f−w curve is transformed to the $f-w^{2}$ curve, the equilibrium path exhibited an approximately linear relationship, as shown by the dotted line in Figure 12b. Thereafter, the intersection points between the straight line extending from the equilibrium path and the f axis can be defined as the approximate critical buckling strength. The ultimate buckling strength after the critical buckling strength can be defined as the post local buckling strength of these flanges.

### 4.2. Estimation of Inelastic Local Buckling Strength of Thin-Walled UHPC Flanges

The characteristics of inelastic local buckling behavior and the strength of UHPC flanges were investigated based on the GMNIA analysis. The CDP material model was used to simulate the nonlinear material model of UHPC. Additionally, the elastic-perfectly plastic material model was applied to the reference flange models, which represent steel flanges with equal yield strength under compression and tension.

The flange models analyzed in this study were supported by a hinge and fixed boundary conditions, including web constraints. If the web is thick, it is similar to a fixed support condition, which does not allow displacement or rotation. If the web is considerably thin, it allows an almost free rotation, similar to the hinge boundary condition. In practical scenarios, steel I-girder flanges supported by one web are generally located between the hinge and fixed boundary conditions, as indicated in Equation (7). However, in the case of UHPC flanges, the web constraints can be considered as fixed boundary conditions, because thick UHPC webs with shear rebars are preferred to secure sufficient shear resistance capacity. In this study, the two boundary conditions were compared for the purpose of theoretical investigations. The analysis results were evaluated in terms of the critical buckling strength and post-buckling behavior of the UHPC flanges.

#### 4.2.1. Axial Compressive Strength and Out-of-Plane Displacement Relationships

- Elastic-Perfectly Plastic Model: Steel Flanges

The elastic-perfectly plastic material model (hereinafter, plastic model) was considered as a conventional material model to simulate the local buckling phenomenon for the steel flanges. The plastic material models have equal yielding strengths under compression and tension; these models further characterize the excellent ductility induced by the plateau with a high strain rate after yielding. Here, the plastic material model was used as a reference model to demonstrate the conventional local buckling phenomenon of a flange with the properties of steel. In the following section, the local buckling behaviors of the UHPC flanges and the steel flanges are compared.

**Figure 13:**
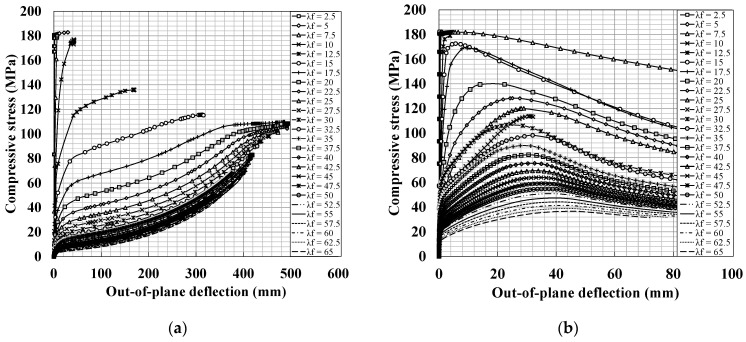
Relationship between compressive stress and out-of-plane deflection for the steel flanges modeled using the elastic-perfectly plastic model according to slenderness ratio ($\lambda_{f}$): (**a**) hinge support condition and (**b**) fixed support condition.

Figure 13 shows the compressive stress and out-of-plane deflection relationships of the flanges, as simulated by the plastic model. As plotted in Figure 13, typical critical buckling and post-buckling behavior that demonstrated the same trend (Figure 12a) were observed. The critical and post-buckling strengths increase as the slenderness ratio decreases. The post-buckling strength of the plastic model was considerably higher than the measured approximate critical buckling strength when the slenderness ratio exceeded approximately 12.5 and 22.5 for the hinge and fixed boundary conditions, respectively. If the slenderness ratio is below these values, the material nonlinearity becomes a controlling factor instead of geometrical nonlinearity. In these cases, as it is difficult to distinguish between critical buckling and post-buckling states, the maximum strength can be considered as the ultimate strength of the flange.

- CDP Model: UHPC Flanges

**Figure 14:**
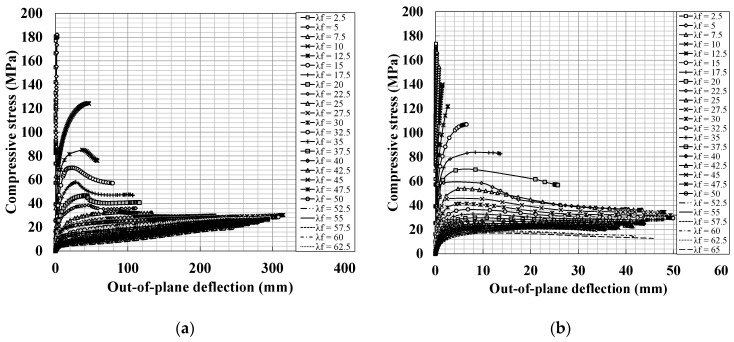
Relationship between compressive stress and out-of-plane deflection for UHPC flanges modeled using the CDP material model according to slenderness ratio ($\lambda_{f}$): (**a**) hinge support condition and (**b**) fixed support condition.

The local buckling strength of UHPC flanges was investigated based on the CDP material model. In the analysis, CDP material models with different failure strengths under compression and tension were considered; the compressive and tensile strengths were considered as 180 MPa and 8.7 MPa, respectively. The nonlinear compression and tension constitutive relations were considered as listed in Table 4 and plotted in Figure 9. Figure 14 shows the compressive stress and out-of-plane deflection relationships. Post-buckling behavior, which demonstrates a trend similar to that shown in Figure 12a, was observed in the FE analysis results. However, considerable reductions in the post-buckling strength were observed.

The post-local buckling strength of the flanges was measured, except in the case of low slenderness ratio (approximately less than 25 and 50 for the hinge and fixed support conditions, respectively). The post-buckling strength of the CDP model, as shown in Figure 14, decreased drastically compared to that of the elastic-perfectly plastic model (Figure 13), regardless of the boundary conditions. This implies that the post-buckling behavior of UHPC flanges had a significant effect on material yielding, particularly the tensile strength, as compared with fully plastic material models, such as those for steel flanges. This is because the tensile strengths of UHPC materials were lower than their compressive strengths, leading to crack behavior during the post-buckling behavior of UHPC flanges.

#### 4.2.2. Inelastic Local Buckling Strength of UHPC Flanges

**Figure 15:**
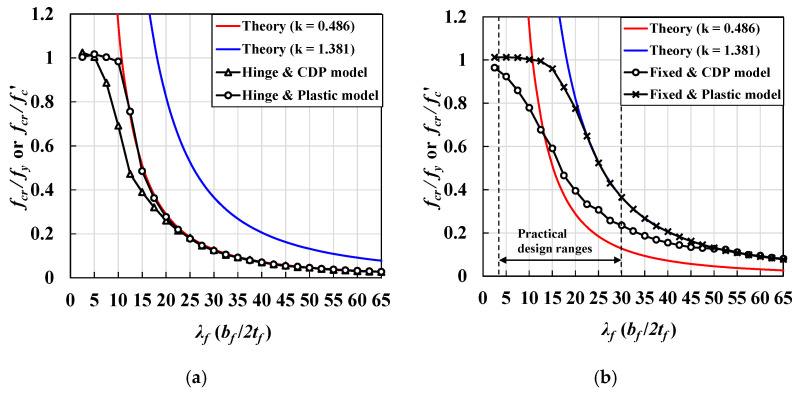
Comparisons between theoretical local buckling strength and flange local buckling strength based on GMNIA according to slenderness ratio and boundary conditions: (**a**) hinge support conditions and (**b**) fixed support conditions.

Figure 15 shows a comparison of the theoretical local buckling strength calculated using Equations (6) and (8) with the critical and inelastic buckling strengths obtained from the GMNIA under two support conditions (Figure 15). The critical and inelastic buckling strengths from the GMNIA were obtained based on the compressive stress and out-of-plane deflection relationships in Figure 13 and Figure 14. The critical buckling strength and inelastic buckling strength can be distinguished based on the intersection between theoretical buckling strength curves and the FE analysis data points, as shown in Figure 15.

Considering the hinge support condition, the approximate critical buckling strength of the steel flanges, obtained using the plastic model, was similar to the theoretical buckling strength, as compared in Figure 15a. However, the approximate critical bucking strength of the UHPC flanges was lower than the theoretical value, when the slenderness ratio was less than 25.

Similarly, for the fixed condition, the inelastic local buckling strength of the steel flange reduced when the slenderness ratio was between 15 and 22.5, whereas that of the UHPC flanges, as determined using the CDP material model, exhibited a nonlinear relationship, with a significant reduction in strength for a slenderness ratio of 2.5–50. Thus, the intersection point distinguishing the critical and inelastic buckling strengths of UHPC flanges could attain a higher slenderness ratio than that of steel flanges. It was noted that, under the fixed condition, the effects of material nonlinearity were more critical than those under the hinged condition.

Additionally, considering practical deck geometric conditions supported by a thick web, realistic deck constraints can be approximately assumed as the fixed support conditions with a slenderness ratio of 5–30 for the flanges, as reported in the literature [22]. Here, realistic buckling modes may be assumed as inelastic local buckling rather than elastic local buckling within the practical design ranges, as shown in Figure 15b. Figure 15b does not confirm that the practical UHPC bridges could fail with local buckling. It is because the practical bridge deck is usually designed based on effective width-based design approaches that may provide conservative deck designs. Additionally, conventional UHPC concrete decks are stiffened to achieve adequately prestressed systems in which the stiffened bridge decks possess a higher local buckling coefficient than unstiffened flange sections. However, it should be noted that the possibility of the local buckling phenomenon could exist not only for steel structures but also for the UHPC bridges within the design ranges. Thus, research on the local buckling phenomena could be necessary to achieve more reasonable, safe, and careful design practices for the application of thin-walled UHPC bridge systems.

## 5. Evaluation of FEA Approaches Based on Test Data

### 5.1. Summary of the Experimental Test in the Literature

**Figure 16:**
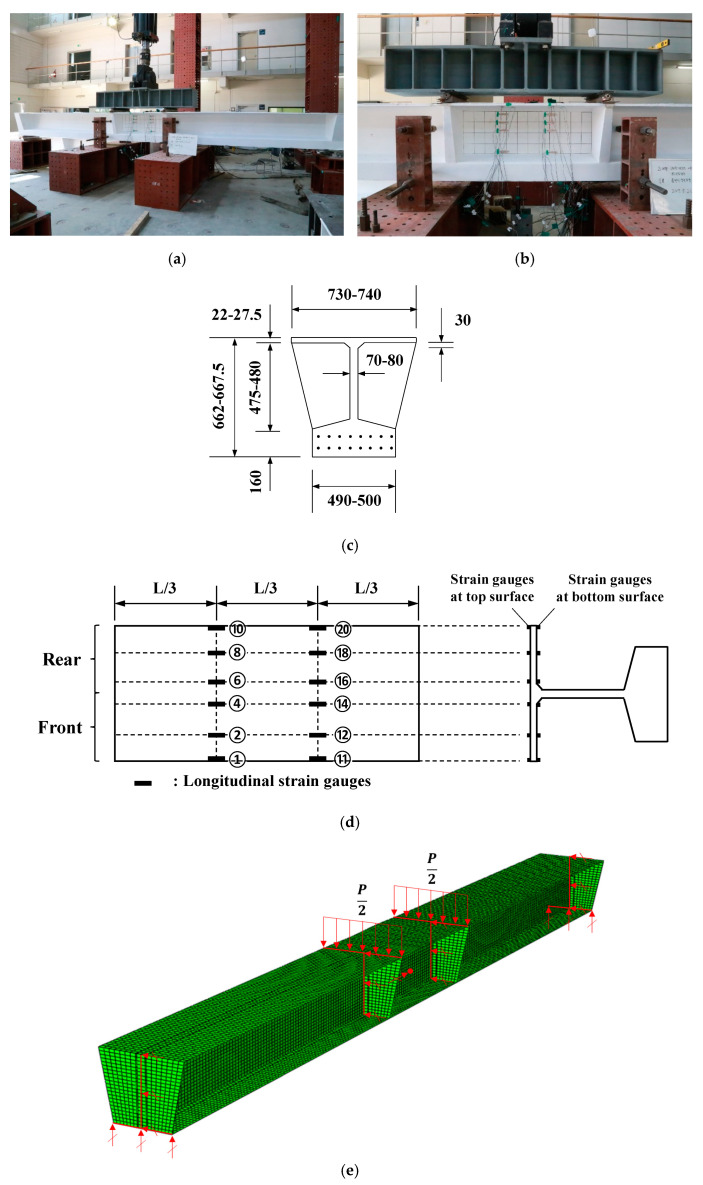
Test specimens and test set-up in literature. (**a**) Illustration of test setup, (**b**) test zone of the specimens, (**c**) dimensions of cross-section of flanges at test zone, (**d**) longitudinal strain gauge locations of flanges in the test zone, and (**e**) an example of the established three-dimensional FEA model (test specimens conducted from reference [23]).

Lee [23] investigated the local buckling behavior of UHPC flanges subjected to pure compression based on experimental tests (Figure 16a). The girder specimens with a three-different thickness of thin-walled flanges were made and four-point bending loading was applied using spreader beams to introduce pure compression of the flanges on the test zone (Figure 16b). The specimens have 22–27.5 mm flange thickness with 730 mm and 740 mm width to induce flange local buckling (Figure 16c). UHPC concrete used for producing the specimens has 162 MPa of compressive strength, and 47,166 MPa of young’s modulus, which was determined by axial compression material tests. For tensile behavior, three-point bending tests were conducted to measure crack width (w). Using the measured crack displacement, crack width at tensile strength ($w_{u}$) and crack width at zero strength beyond the tensile strength ($w_{lim}$) were determined. Tensile strength was determined by $7.8\sqrt{f_{c}^{\prime }},$ which is a lower boundary strength suggested by Graybeal [3]. The summary of the material test results was listed in Table 5. To earn critical strain rates when the flanges of test specimens buckle, longitudinal strain values of the top and bottom side of flanges were measured during the tests (Figure 16d). Three-dimensional finite element analysis models for evaluating the test specimens considering the local buckling behavior were established in their research. Four-point loadings and simply supported boundary conditions with lateral supports to prevent overturning of the specimens used in the test were applied in the presented FEA models to simulate similar structural behavior of test specimens as possible as shown in Figure 16e.

**Figure 17:**
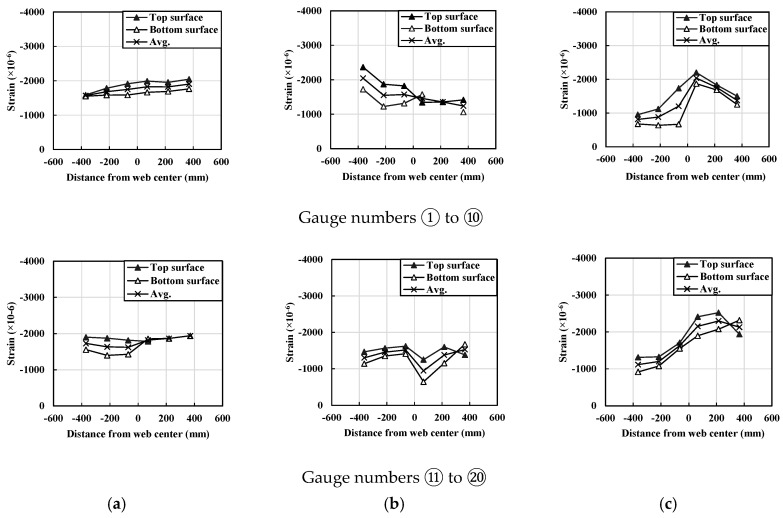
Compressive strain distributions in the longitudinal direction of UHPFRC flanges: (**a**) specimen #1, (**b**) specimen #2, and (**c**) specimen #3 (data from reference [23]).

In order to determine the maximum strength of the compression flange sections, strain values along the flange width were measured as describe the location of strain gauges in Figure 16d. The strain gauges were installed on the top and bottom surfaces of the flange section as described in Figure 16d. Figure 17 shows strain values of both top and bottom surfaces of the flanges along the flange width were measured at ultimate strength state during the tests. Based on the strain values, the average maximum compressive strain was determined. The maximum average compressive strain ($\varepsilon_{avg}$) induced by compressive stress was determined as listed in Table 6. Additionally, design moment strength ($M_{design}$) and test strength ($M_{test}$) due to the local buckling were also provided in Table 6. From the established 3D FEA models corresponding to the test specimens, normalized compressive stresses ($f_{FEA}/f_{c}^{\prime }$) considering fiber orientations factor ($K_{f}$) due to material uncertainty during concrete casting were also presented in Table 6. More detailed information of design, test, and FEA values were presented in the literature [23].

### 5.2. Comparisions between FEA and Test Data

**Figure 18:**
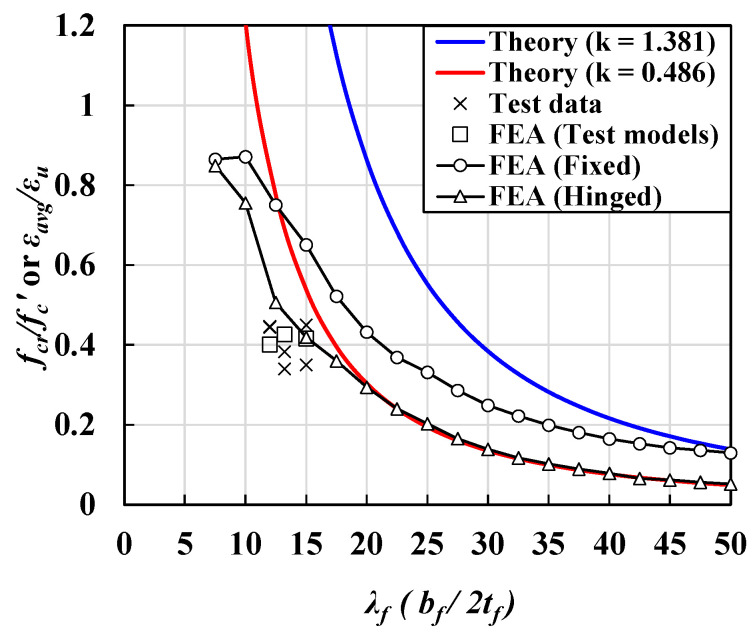
Comparisons between presented FEA results and test data in the literature (test data from reference [23]).

Figure 18 shows a comparison of maximum average strain values from the test data and maximum stress values based on flange models established in this study. Additionally, the maximum stress values based on 3D FEA models corresponding to the test specimens presented in the literature [23] also compared. The finite element analysis was conducted based on the material test data in Table 5. Maximum stress values according to the slenderness ratio were analyzed based on the analysis results simulated with the same analysis approaches presented in the previous section. Based on measured data from the literature, 3 mm of initial imperfections were applied. As shown in Figure 18, the test data shows similar trends compared with finite element analysis results. The test data were located at lower parts of the graph than the analysis results of fixed and hinge boundary conditions. All test data shows better correlations with hinged boundary conditions. Considering the uncertainty of the material properties of concrete and realistic boundary conditions, the test results may show good correlations with the presented finite element analysis.

## 6. Characteristic of Local Buckling Behavior of Thin-Walled UHPC Flanges

### 6.1. Inelastic Local Buckling Behavior of UHPC Flanges

**Figure 19:**
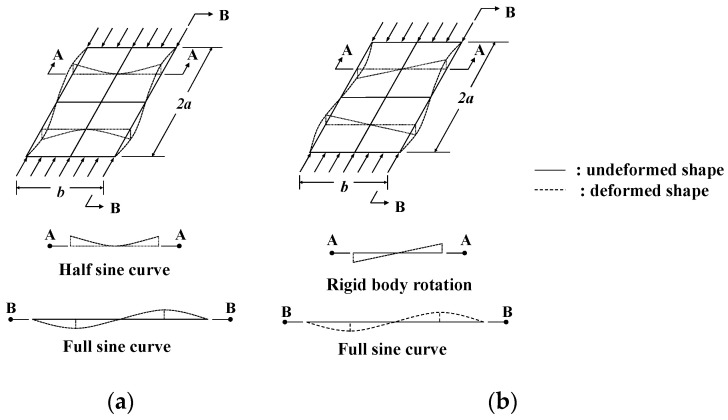
Comparison of buckling mode shapes between (**a**) fixed support condition, and (**b**) hinge support condition.

The buckling mode of the fixed support condition simultaneously demonstrated a conventional sine curve along the longitudinal direction (B-B line) with a half-sine curve along the transverse direction (A-A line) (refer to Figure 19a). However, in the case of hinged support conditions, a conventional sine curve along the longitudinal direction (B-B line) was observed, along with rigid body rotation along the transverse direction (A-A line) (refer to Figure 19b). These differences between the buckling modes along the transverse directions may result in considerable local bending under high bending stresses, particularly in the case of the fixed support condition. Due to the local bending behavior with higher curvatures, the material nonlinearity effects under fixed support conditions have a greater influence on the inelastic local buckling strength of UHPC flanges. Further detailed evaluations of these aspects are presented in the following sections.

### 6.2. Estimation of Local Buckling Behavior of UHPC Flanges

**Figure 20:**
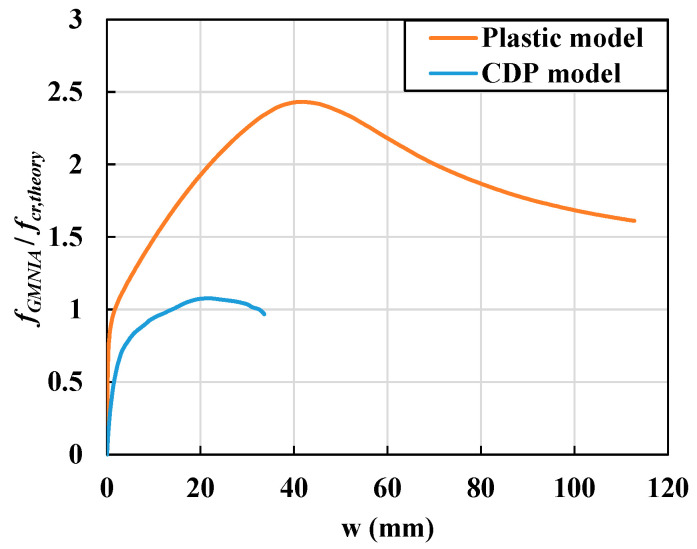
Relationship between normalized local buckling strength and slenderness ratio under the fixed support condition when $\lambda_{f}$ = 55.

In this section, local buckling behavior is discussed in terms of the local bending stresses and strains along with the longitudinal and transverse directions for the two different models, namely the CDP and plastic material models. The CDP model represents the behavior of UHPC flanges, while the plastic material model represents conventional steel flange sections and serves as the reference model. This comparison is conducted to investigate the effects of the nonlinear material models and, CDP model on the local bending and buckling behavior of UHPC flanges. Figure 20 shows the relationship between the normalized compressive stress and out-of-plane displacement of the flanges according to the two different material models. The flange with a slenderness ratio of 55 was selected and fixed boundary conditions were adopted. Out-of-plane displacement was measured at the node where maximum displacement occurred, and the compressive stresses were calculated by dividing the compressive loads acting on each end of the flange with the cross-sectional areas.

The plastic material model exhibited conventional post-buckling behavior, where buckling strengths gradually increased with large out-of-plane deformation after the critical buckling strength. However, in the case of the CDP material model, the initial stiffness and ultimate strength of the flange were significantly lower than those of the plastic material model. To further understand such local buckling behaviors, the local bending stresses in the flange section were evaluated using strain and stress diagrams along the thickness direction for each loading phase determined by the material yielding states.

**Figure 21:**
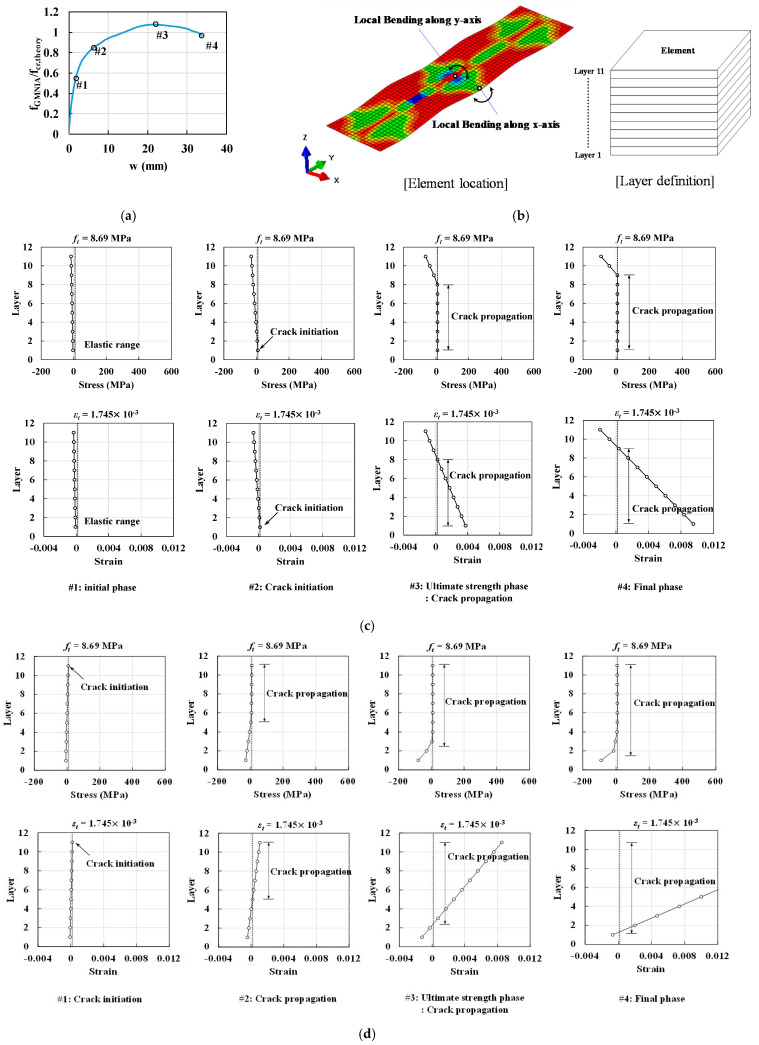
Comparisons of compressive stress and strain for UHPC flanges, modeled using the CDP material model, according to loading phases: (**a**) definition of loading sequence, (**b**) definition of local bending location and element layers, (**c**) normal stress distribution of local bending along the *X*-axis, and (**d**) normal stress distribution of local bending along the *Y*-axis.

**Figure 22:**
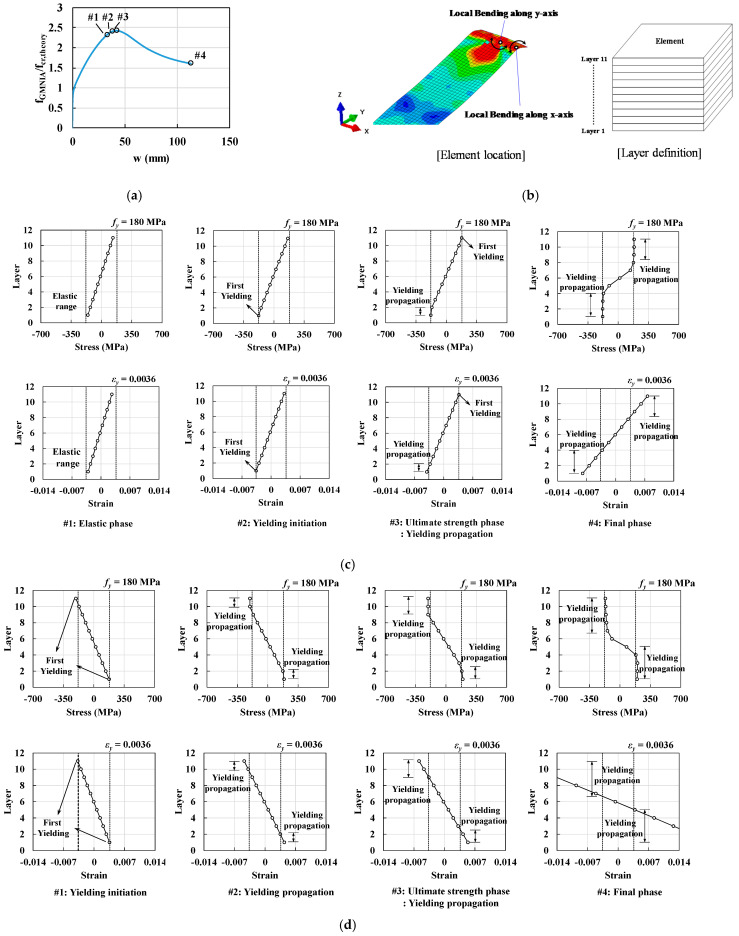
Comparisons of compressive stress and strain for ordinary steel flanges, modeled using the plastic material model, according to loading phases: (**a**) definition of loading sequence, (**b**) definition of local bending location and element layers, (**c**) normal stress distribution of local bending along the *X*-axis, and (**d**) normal stress distribution of local bending along the *Y*-axis.

To estimate the local bending behavior of UHPC and steel flanges, the stress and strain states at each loading phase were classified into four different phases, as shown in Figure 21 and Figure 22. The notations #1–#4 in Figure 21 and Figure 22 denote (1) the first crack or yielding initiation phase in the longitudinal axis near the centerline, (2) the second crack or yielding initiation phase in the transverse axis near the flange tips, (3) the ultimate buckling strength phase after the crack or yielding propagation, and (4) the final phase after the analysis, respectively.

In the case of the CDP material model, the first crack occurred due to the transverse stress and strain along the web line (refer to phase #1 in Figure 21d). After the initiation of the crack, the stiffness of the compressive stress and out-of-plane displacement curve exhibited a decline (refer to phase #1 in Figure 21a), because the effective compressive area started decreasing due to crack propagation. Second, the strain along the longitudinal direction resulted in a crack at the flange tips (refer to phase #2 in Figure 21c); these cracks propagated via the transverse stress along the web line, leading to a significant reduction in stiffness (refer to phase #2 in Figure 21d). Third, crack propagation increased along with the longitudinal and transverse directions until the ultimate strength state was reached (refer to phase #3 in Figure 21a–d). A further increase in strength was not observed owing to the decrease in the effective compressive area, which was caused by changes in the neutral axis of the flanges due to crack propagation (refer to phase #4 in Figure 21a–d).

The stiffness and ultimate strength of the reference steel flange simulated using the plastic material model under fixed boundary conditions were significantly different from those of the UHPC flanges, which were modeled using the CDP material model. For the plastic material model, the first yield along the web line, induced by the transverse stress and strain components, occurred immediately before the ultimate strength state (refer to phase #1 in Figure 22d). After the initiation of yield, stiffness declined marginally, which is reflected by the compressive stress and out-of-plane displacement curve (refer to phase #1 in Figure 22a). Subsequently, the compressive strain along the longitudinal direction at flange tips reached the yield strain (refer to phase #2 in Figure 22c). Yielding in the compression and tension sides of the flange progress; thereafter, transverse stress occurred along the web line owing to the decline of stiffness (refer to phase #2 in Figure 22d). Furthermore, after the propagation of the yield along the longitudinal direction at the flange tips, the ultimate was attained (refer to phase #3 in Figure 22a,c). Compressive stress decreased as the yield propagates along the longitudinal direction (refer to phase #4 in Figure 22a–d).

**Figure 23:**
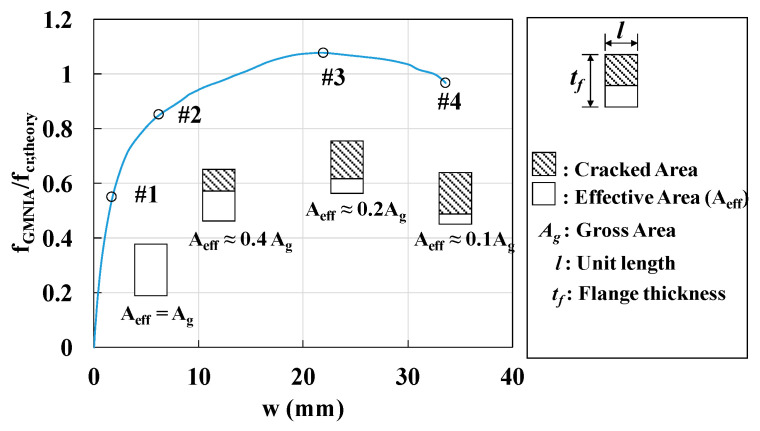
Variation of effective area during each loading sequences.

The FE analysis results indicated that there were significant differences in the local buckling and local bending behaviors of the CDP material and plastic material model within inelastic ranges. These differences were attributed to the considerably lower tensile strength of UHPC, as compared to that of conventional steel flanges. In the UHPC flanges, initial cracks developed near the centerline; a reduction in stiffness, caused by the loss of the effective cross-section ($A_{eff}$) and the neutral axis changes, was observed due to crack propagation, as illustrated in Figure 23.

For the plastic material model, although yielding was initiated near the centerline of the flange, the stiffness and strength reduction were not significant, because the gross cross-sectional area remains effective for the local bending behavior. This may further account for the tensile capacity of UHPC, which was approximately 20 times lower than that of the plastic models. Additionally, the plastic models did not exhibit strength reduction due to their ductility.

The second reason is the different failure mechanisms between UHPC and steel flanges. For steel flanges, which are represented by the plastic material model, the main strength reduction under inelastic local buckling behavior was induced by compression yielding at the flange tips (refer to Figure 22c,d). Thus, compression steel flanges did not exhibit strength reduction until they reached compression yielding. In contrast, the controlling failure modes in UHPC flanges were mainly tensile fractures caused by local bending and not compression-induced failure modes. Therefore, the post-buckling strength of UHPC flanges was particularly susceptible to local failure due to the cracks in the tension side of the flanges. Alternatively, the local failure of the plastic material model was mainly caused by the initiation of compression yielding. Thus, the tensile strength of UHPC can be considered as a major design parameter on the inelastic local buckling strength of UHPC flanges.

## 7. Conclusions

In this study, theoretical elastic and inelastic local buckling strengths, and behaviors of UHPC flanges were evaluated using the GMNIA. Parameters affecting the inelastic local buckling strength of UHPC flanges, including the tensile strength, and boundary conditions, were evaluated and compared with those of plastic material models with equal yield strengths under compression and tension. The important characteristics of UHPC flanges thus identified are presented as follows:

- The elastic buckling strength of UHPC flanges was affected by boundary conditions and Poisson’s ratio. Although the UHPC flanges exhibited a higher elastic buckling coefficient than the steel flanges, the buckling strength of UHPC flanges possessed ¼ times lower values than that of the steel flanges.
- Nonlinear finite element analysis strategies to simulate the local buckling behavior of UHPC flanges were established based on geometric and material nonlinear analysis with imperfections (GMNIA). It was verified based on test data conducted in the literature. The finite element analysis results and test data show good correlations in accordance with maximum average strain values along with the longitudinal directions.
- The post-buckling strength of UHPC flanges was found to be considerably lower than that of the reference steel flanges, under both hinged and fixed support conditions. Particularly, UHPC flanges with fixed support conditions were susceptible to a considerable reduction in post-buckling strength because these flanges underwent local bending and tensile cracks caused by simultaneous longitudinal and transverse stress components.
- Considering practical deck designs, local buckling of the UHPC flange deck could potentially exist within the design range with severe cases. In these cases, realistic buckling modes may be considered as the inelastic local buckling instead of elastic local buckling.

## Figures and Tables

**Table 1:** An ultra-high-performance concrete (UHPC) mix design example by weight.

W/B	Cement	Fine Sand	Silica Fume	Glass Powder	Water Reducer	Steel Fiber (Volume)
0.18	1	0.92	0.25	0.25	0.0108	0.22–0.31

**Table 2:** Elastic flange buckling coefficient (k) according to the Poisson’s ratio (μ).

Cases	E	μ	k	
			Hinge Support	Fixed Support
Steel	205,000	0.3	0.426	1.289
UHPC	49,790	0.2	0.486	1.381

**Table 3:** Parameters of the GMNIA analysis.

Cases	E (MPa)	μ	$f_{c}^{\prime }$ or $f_{y}$ (MPa)	$f_{t}$ or $f_{y}$ (MPa)	$b_{f}$ (mm)	$\lambda_{f}$
Steel	205,000	0.3	180	180	1000	2.5–65 (26 cases at 2.5 intervals)
UHPC	49,790	0.2		8.7		

**Table 4:** Material properties for tensile behavior of UHPC applied to the CDP model.

E (MPa)	$f_{c}^{\prime }$ (MPa)	$\varepsilon_{u}$	$f_{t}$ (MPa)	$w_{u}$ (mm)	$w_{lim}$ (mm)
49,790	180	0.0042	8.67	0.3	5.3

**Table 5:** Material properties based on test data from the literature [23].

E (MPa)	$f_{c}^{\prime }$ (MPa)	$\varepsilon_{u}$	$f_{t}$ (MPa)	$w_{u}$ (mm)	$w_{lim}$ (mm)
47,166	162	0.00396	8.24	0.22	6.43

**Table 6:** Comparisons between average strain ($\varepsilon_{avg}$) and maximum material strain ($\varepsilon_{cu}$) (data from reference [23]).

Specimens	$b_{f}$ (mm)	$t_{f}$ (mm)	$\lambda_{f}$	$M_{,design}$	Test							FEA
					$M_{test}$	$\frac{M_{test}}{M_{design}}$	$\varepsilon_{u}$	$\varepsilon_{avg}$		$\varepsilon_{avg}/\varepsilon_{u}$		$f_{FEA}/f_{c}^{\prime }$ ($K_{f}$ = 1.7)
								Gauges ① to ⑩	Gauges ① to ⑩	Gauges ① to ⑩	Gauges ① to ⑩	
Specimen #1	730	27.5	12	2221	1103	0.497	0.00396	0.001766	0.00176	0.446	0.444	0.401
Specimen #2	730	25	13.2	2056	1005	0.489	0.00396	0.001516	0.001346	0.383	0.34	0.426
Specimen #3	740	22	15	1984	1111	0.56	0.00396	0.001387	0.001779	0.35	0.45	0.416

## Data Availability

The data presented in this study are available on request from the corresponding author.
